# Dental biofilm acidogenicity induced by pediatric oral medications: a double-blind randomized clinical trial

**DOI:** 10.1590/1807-3107bor-2024.vol38.0107

**Published:** 2024-11-08

**Authors:** Karla Pinheiro de Alencar, Daniel Fernandes Peixoto, Fábio do Nascimento Máximo, Isabela Albuquerque Passos Farias, Fábio Correia Sampaio

**Affiliations:** a Universidade Federal da Paraíba – UFPB, Laboratory of Oral Biology, João Pessoa, PB, Brazil.; b Universidade Federal da Paraíba – UFPB, Department of Restorative Dentistry, João Pessoa, PB, Brazil.; c Universidade Federal da Paraíba – UFPB, Department of Clinical and Social Dentistry, João Pessoa, PB, Brazil.

**Keywords:** Dental Plaque, Administration, Oral, Dental Caries

## Abstract

The aim of this study was to evaluate *in vivo* dental biofilm acidogenicity induced by nine long-term pediatric oral liquid medications (OLMs). A double-blind crossover randomized clinical trial was conducted with 12 individuals aged 18 to 22 years who had good oral hygiene (OSI < 1.1) and a DMFT index of less than 12. Each participant was exposed to nine OLMs and a 10% sucrose solution (positive control) as part of the crossover design. The pH of the dental biofilm was measured with a Beetrode® microelectrode at 0, 5, 10, 15, 20, 25, and 30 min. Statistical analysis was performed to determine the minimum pH and the area under the curve (AUC). One-way ANOVA was utilized, and the significance level was set at 0.05. Pediatric OLMs caused a sucrose-like decrease in biofilm pH, regardless of therapeutic class (p > 0.05). The mean ± standard deviation of the AUC ranged from 16.26 ± 11.59 (cetirizine) to 39.22 ± 20.81 (azithromycin), with no statistically significant difference compared to sucrose (25.22 ± 6.97) (p > 0.05). The findings suggest that pediatric OLMs contribute to dental biofilm acidogenicity, with a more pronounced effect induced by medications used for respiratory diseases and also by antibiotics.

## Introduction

The pH of the intraoral environment causes demineralization of both dental surfaces and subsurfaces, leading to the development of caries and dental erosion.^1-3^ The average prevalence of dental caries is 46.2% in deciduous teeth and 53.8% in permanent teeth among children.^4^ Likewise, the global prevalence of erosive tooth wear ranges from 30% to 50% in deciduous teeth and from 20% to 45% in permanent teeth.^5^

To reduce the prevalence of demineralization, it is essential to control dental biofilm and monitor the intake of carbohydrates and acidic liquids, either from the diet or from oral medications.^1-3,6^

Oral liquid medications (OLMs) are the first-line treatment for children. Therefore, acceptance of these formulations by the children is the first step towards successful therapy.^7^ To make OLMs more palatable for pediatric patients, the pharmaceutical industry uses sugars, mainly sucrose, in large quantities, in their formulations. Sucrose is included in almost all formulations designed for children.^8^

In addition, the low endogenous pH of OLMs can predict their cariogenic and erosive potential.^9^ However, pH alone is not a decisive factor for dental demineralization.^10^

A previous *ex vivo* clinical trial found a decrease in biofilm pH comparable to that caused by sucrose after exposure to two analgesics,^11^ an antihistamine, and another analgesic.^8^ However, no effect of OLMs was observed on *in vivo* biofilm pH, which can be attributed to the protective effect of saliva.^12^

The paucity of studies in the literature, the high prevalence of caries and dental erosion in the world population, and the chronic and frequent exposure of children with chronic diseases to OLMs ^13^ underscore the need to assess whether OLMs can potentially lower the pH of dental biofilm as occurs with 10% sucrose.

Accordingly, the aim of this study was to evaluate *in vivo* dental biofilm acidogenicity induced by nine long-term pediatric OLMs. The study also sought to establish the dose-response relationship of OLMs at different sucrose concentrations (control group) in the biofilm pH curve, evaluate the pH variation over time, and determine the possible antimicrobial effect of some of these medications.

## Methods

### Study design, ethics, and recruitment of volunteers

This was a double-blind crossover randomized clinical trial. The study participants switched medications after a washout period, enabling each participant to serve as their own comparator. The study was conducted at the Cariology Outpatient Clinic of the Federal University of Paraíba, Brazil.

This study followed the ethical recommendations set by the Declaration of Helsinki and Resolution 510/2016 of the Brazilian National Health Council. The protocol was approved by the Ethics and Research Committee of the Federal University of Paraíba (protocol no. 0329/11), and it was registered with the Good Clinical Practice Network (NCT00723515).

The study included 12 healthy Brazilian male and female adults aged 18 to 21 years, who had good oral hygiene (simplified oral hygiene index < 1.1) and a DMFT (decayed, missing, and filled teeth) index of less than 12. Individuals with comorbidities, those undergoing antibiotic or other antimicrobial therapy during the study period, those receiving orthodontic treatment, and smokers were excluded from the study.

### Medication selection

Oral liquid medications (n= 9) from different therapeutic classes were purchased at local stores in the city of João Pessoa, Brazil (Table 1). The selected OLMs had sucrose concentrations between 2.23 ± 0.42% (Anemifer®) and 59.68 ± 3.46% (Asmofen®), and endogenous pH of 2.3 ± 0.01 to 10 ± 0.02 (Azi®).^14^

**Table 1:** Characteristics of nine long-term pediatric oral liquid medications.

Therapeutic class	Commercial name	Formulation	Manufacturer
Nutritional	Anemifer	Syrup	Pharmascience
	Folacin	Solution	Otivus
Anti-histamine	Asmofen^®^	Syrup	Teuto
	Zetalerg^®^	Solution	Uci-farma
Antibiotic	Keflaxina	Suspension	Hexal
	Uni Amox	Suspension	União química
	Azi	Suspension	Sigma Pharma
Corticosteroid	Dexaglós	Elixir	Belfar
	Celestone	Elixir	Schering-Plough

### Randomization, Intervention, and outcome

Twelve participants per group were randomly assigned to the investigated medications through computerized random number generation.

The order of dental biofilm exposure to OLMs was determined randomly via computerized random number generation. pH measurements were taken in the afternoon following a 24-hour toothbrushing-free period and a 2-hour fasting period to control for potential influences on acidogenicity.^15^

Next, 2 mL of medication or 10% sucrose was dripped onto the proximal surface of the lower anterior tooth, and excess liquid was wiped away with a cotton swab to prevent absorption by the mucosa. A Beetrode® microelectrode (WPI Inc., Sarasota, USA) connected to a potentiometer (Orion 230 A) was used to measure the pH of dental biofilm *in vivo* at seven time points: 0 (initial pH), 5, 10, 15, 20, 25, and 30 min after exposure to the solutions.

The participant dipped one of their fingers into the KCl solution (3 M). The system was calibrated with standard pH solutions of 4.0 and 7.0 before each session. The medication was changed every two weeks among all participants.

### Statistical methods

An analysis was performed to verify statistically significant differences between minimum pH and the AUC for each OLM and between the OLM and the positive control. The AUC was calculated to verify pH recovery, where higher values corresponded to larger areas and slower pH recovery. Normal distribution was confirmed by the Shapiro-Wilk test, and the one-way ANOVA was used with a statistical significance level of 5%.

## Results

The OLMs exhibited pH values below the critical level (pH = 5.5), except for folic acid (pH = 5.97) (Table 2).

**Table 2:** Minimum pH (mean), AUC and standard deviation values obtained for each pediatric long-term use medicines for children.

Variables	Minimum pH (mean)	Standard deviation	AUC^a^	Standard deviation
Folic acid	5.97	0.24	22.91	6.35
Ferrous sulfate	5.00	0.59	34.91	2.79
Cetirizine	5.15	0.40	16.26	11.59
Ketotifen	5.28	0.75	22.19	19.64
Betamethazone	5.33	0.89	26.30	17.17
Dexamethazone	5.45	0.36	20.50	9.37
Cephalexin	5.37	0.55	27.77	13.06
Amoxicilin	5.47	0.87	37.06	4.16
Azithromycin	4.71	0.42	39.22	20.81
Sucrose 10%	5.53	0.50	25.22	6.97

a Area under curve; One-way Anova; p-values > 0.05.

Figure shows the changes in pH of the dental biofilm after *in vivo* exposure to the medications. Two medications from the nutritional group (Figure a) showed a pH value below the critical level (pH = 5.5), significantly lower than those observed for sucrose (positive control).

**Figure:**
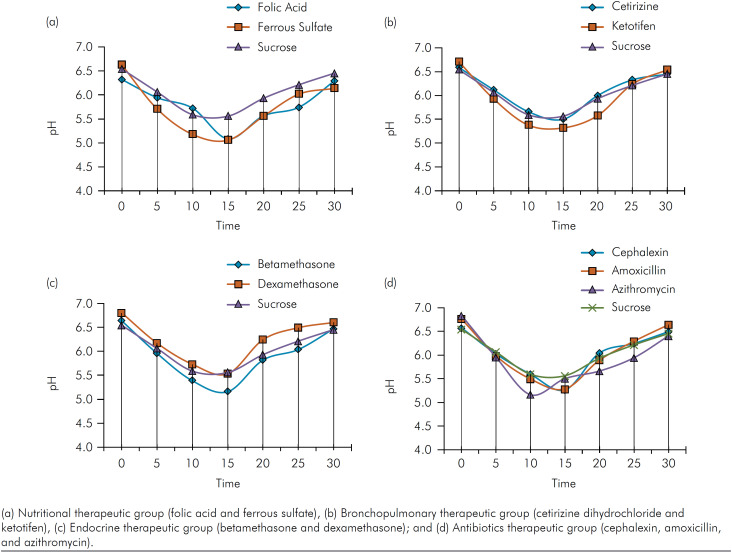
Mean pH of dental biofilm exposed to pediatric oral medications over a 30-minute period.

(a) Nutritional therapeutic group (folic acid and ferrous sulfate), (b) Bronchopulmonary therapeutic group (cetirizine dihydrochloride and ketotifen), (c) Endocrine therapeutic group (betamethasone and dexamethasone); and (d) Antibiotics therapeutic group (cephalexin, amoxicillin, and azithromycin).

OLMs from the respiratory group (cetirizine and ketotifen) showed a minimum pH close to that of the control, demonstrating that ketotifen reached a pH below the critical level (Figure b). The medications from the endocrine group (betamethasone and dexamethasone) (Figure c) exhibited similar behavior to that of the respiratory group, and betamethasone reached a pH below the critical level. Finally, the antibiotics group (Figure d) had minimum pH values below the critical level and lower than those of sucrose, with azithromycin showing the lowest pH in the group.

Biofilm pH values decreased up to 15 min after exposure to folic acid, ferrous sulfate, cetirizine, ketotifen, betamethasone, dexamethasone, cephalexin, and amoxicillin. The biofilm exposed to azithromycin showed a pH decrease in just over 10 min, with the pH graph showing an upward trend thereafter.

## Discussion

This study evaluated the pH of dental biofilm exposed *in vivo* to nine chronic-use OLMs, considering the effect of saliva film thickness and buffering capacity on biofilm pH.^12,16^ In addition, all pH measurements were performed in the afternoon to avoid variations in the circadian rhythm of salivary flow rate among participants.

Our *in vivo* results showed that nine of the tested OLMs were acidogenic, leading to an immediate and prolonged decrease in pH. This finding is consistent with those of other *ex vivo* studies involving oral medications^8,11^ and infant milk formulas.^17^ No difference was observed in relation to 10% sucrose, with a similar response despite the higher carbohydrate concentration in OLMs.^14,18^ This is probably because the average sucrose concentration in OLMs is 31.76%, ranging from 2.23% to 65.01%, according to a previous study.^14^

Furthermore, higher sucrose levels in the dental biofilm can boost the competitiveness of *Streptococcus mutans* within the multispecies biofilm, possibly rendering the biofilm more cariogenic.^19^

OLMs are complex solutions that contain different ingredients, unlike the sucrose solution.^8^ Thus, inactive ingredients had no effect on pH variation.

Overall, the literature reports *in vitro* studies on endogenous pH and sucrose in pediatric medications and beverages in several countries around the world.^3,8,10,14^ However, it is necessary to evaluate the dose-response of drugs in dental biofilm *in vivo* to assess their cariogenic and erosive potential.

In this study, 10% sucrose was used as the control solution, consistent with Sharma et al.^8^ (pH 5.5 and 24.09 AUC). However, ketotifen showed an endogenous sucrose concentration six times higher than that of the control solution, in line with a previous study,^14^ but promoted a decrease in biofilm pH similar to that of sucrose.

The reduction in biofilm pH is exacerbated by frequent daily use and chronic administration. Research on long-term medications (used for six months or longer) has provided evidence^13^ that prolonged use can cause or accelerate dental demineralization.^3,11^

Given the impact of long-term medication use on oral health, it is important to develop strategies to inform both patients and healthcare professionals, including pediatricians and dentists, about the consequences of using OLMs for long periods. A previous study^11^ found that chewing sugar-free gum for 20 min immediately after taking OLMs can restore biofilm pH. Thus, older children can benefit from chewing gum, while younger children still lack a similarly effective intervention after OLM administration.

A limitation of this study is that it did not evaluate factors that can modify the acidogenic potential of drugs, such as retention in the mouth, physical form, protective effect of inactive ingredients, effect of OLM on bacterial colonization, and the amounts and type of carbohydrate composition.^8^ While biofilm growth time was the same among participants, there may be differences in biofilm thickness and pH changes depending on biofilm volume.^20^

All participants had good oral hygiene, which suggests that the biofilm was not In a previous study,^15^ sucrose reduced the biofilm pH of children with and without early childhood caries, but higher pH variation was observed in the caries group than in the caries-free group. Moreover, the biofilm of patients with dental caries had a higher count of *S. mutans*.

In addition, this study was conducted with adult participants. Oral microbiota diversity seems to be higher in adolescents and children than in adults,^21^ and plaque pH responses are less acidic in children than in adults.^22^ This underscores the need for further comparative studies involving both adults and children to assess the effects of OLMs on the microbiota and pathogenic biofilm.

Thus, physicians have to be aware of carbohydrate concentrations in drugs and provide guidance on oral hygiene as part of comprehensive patient care. It is essential that the prescription of sugar-containing medications be accompanied by instructions on proper oral hygiene to prevent the development of dental caries, especially among patients who require long-term use of OLMs.

## Conclusion

Our findings suggest that pediatric OLMs contribute to dental biofilm acidogenicity, especially medications used for respiratory diseases and antibiotics, which have greater acidogenic potential.
